# Apoptotic Effects of a Chimeric Plant Virus Carrying a Mimotope of the *Hepatitis C virus* Hypervariable Region 1: Role of Caspases and Endoplasmic Reticulum-Stress

**DOI:** 10.1007/s10875-012-9676-1

**Published:** 2012-03-06

**Authors:** G. Piazzolla, M. Nuzzaci, A. Vitti, N. Napoli, M. Schiavone, P. Piazzolla, S. Antonaci, C. Tortorella

**Affiliations:** 1 Department of Internal Medicine, Immunology and Infectious Diseases, Section of Internal Medicine, University of Bari, 70124 Bari, Italy; 2 Department of Biology, Plant Protection and Agrobiotechnology, University of Basilicata, Viale dell’Ateneo Lucano, 10, 85100 Potenza, Italy

**Keywords:** Apoptosis, HCV, cucumber mosaic virus, caspases, ER-stress

## Abstract

The role of apoptosis in the persistence of hepatitis C virus (HCV) infection is controversial. Moreover, conflicting data on the modulation of this process by HCV proteins have been provided. We evaluated the susceptibility of peripheral lymphocytes from patients with chronic hepatitis C to apoptosis both spontaneous and after incubation with a chimeric *Cucumber mosaic virus* (CMV) carrying 180 copies of the synthetic R9 mimotope obtained from more than 200 hypervariable region-1 sequences of HCV. Resting T lymphocytes were found to be sensitized to apoptosis as a result of chronic HCV infection. The plant virus-derived vector R9-CMV displayed a strong pro-apoptotic effect associated with activation of both caspase-8 and −9, indicating the involvement of both extrinsic and intrinsic apoptotic pathways. A parallel R9-CMV-mediated activation of endoplasmic reticulum-stress was suggested by the significant induction of BiP/GRP78, GADD153 and caspase-12. These data contribute to define the complex HCV/host interaction, and open new prospects for developing a plant-derived antigen-presenting system to strengthen host defences against persistent pathogens.

## Introduction

The ability of *hepatitis C virus* (HCV) to persist indefinitely in the infected host, together with the current unavailability of a fully effective therapy and of a protective vaccine against this virus, are the main reasons why HCV infection is imposing a growing burden on health systems worldwide. The mechanisms whereby this pathogen establishes persistent infection remain elusive, but it is widely recognized that subversion of host immune responses by HCV might explain the high rate of chronic infections. HCV clearance has, in fact, been correlated with strong and persistent cytotoxic T lymphocyte (CTL) responses directed against multiple HCV epitopes [1–3]. In this context, a chimeric form of the plant virus *Cucumber mosaic virus* (CMV), the so-called R9-CMV [4], genetically engineered to express on its coat protein 180 copies of the synthetic R9 mimotope obtained from more than 200 hypervariable region (HVR)-1 sequences of HCV [5], has been demonstrated to induce a significant enhancement of HCV-specific CTL activities [6, 7].

Modifications of cell apoptosis have been hypothesized to affect HCV persistence and virus-related tissue damage as well, even though it has still to be understood whether the apoptotic process is linked to the clearance or the persistence of HCV infection [8]. Although there is increasing evidence suggesting that liver damage caused by viral infections (i.e. HBV, HCV), autoimmune reactions, toxins and cholestasis is mediated by inappropriate induction of cell apoptosis [9], it is currently assumed that programmed cell death is an important mechanism of host defence against intracellular pathogens and tumorigenesis [10]. Cytotoxic T lymphocyte effectiveness in destroying virus-infected cells or any other cell that poses a threat to the integrity of the organism is strictly related to the CTL ability to trigger the apoptotic process directly or through the mediation of pro-apoptotic cytokines [11]. In this regard, a defective apoptosis has been implicated as a major determinant in the development of hepatocellular carcinoma (HCC) [12]. Moreover, the HCV ability to directly infect immune cells, including peripheral lymphocytes and monocytes, might affect their function and down-modulate apoptotic events, thereby allowing virus chronic replication in target cells [13]. However, the susceptibility of the peripheral immune system to the apoptotic process in the course of chronic HCV infection has not yet been studied in depth.

To date, there is still controversy as regards the apoptotic effect of HCV, also because the genetic heterogeneity of the virus makes it difficult to compare the apoptotic pathways elicited by different HCV genotypes. The role of specific HCV proteins in such a process also remains unclear. The HCV core protein has been widely studied and in vitro it has been shown either to inhibit or to enhance apoptosis induced by several stimuli, depending on experimental conditions and type of cells used [14–17]. Similarly, both anti- and pro-apoptotic effects of HCV NS3, NS4A, NS5A and NS5B proteins have been described [18–21] and few, conflicting data are available on the HCV envelope protein-mediated modulation of programmed cell death [22, 23].

The current study was designed to evaluate whether chronic HCV infection affects the susceptibility of peripheral immune cells to the spontaneous apoptosis process. In addition, we used the plant-derived antigen (Ag)-presenting system R9-CMV to investigate the ability of HVR1-derived peptides to modulate, in vitro, peripheral lymphocyte apoptosis. We found that resting T lymphocytes isolated from patients with chronic hepatitis C were sensitized to apoptosis and that the R9 mimotope, expressed on the CMV surface, exerted a strong pro-apoptotic effect on peripheral lymphocytes. Interestingly, the chimeric virus R9-CMV induced significant levels of both active caspase-8 and −9, suggesting that both the extrinsic and the intrinsic apoptotic pathway contributed to the downstream activation of the R9-CMV-dependent apoptosis execution phase. At the same time, evidence of a R9-CMV-dependent endoplasmic reticulum (ER)-stress activation, closely related to cell apoptosis, was also gained, opening new perspectives on the mechanisms underlying HCV-induced apoptosis.

## Materials and Methods

### Construction of Chimeric Virus and Plant Infection

Two strains of CMV, namely CMV-D and CMV-S, were propagated in *Nicotiana tabacum* cv. Xanthi and purified as described by Lot et al. [24]. A pseudorecombinant CMV-D/S was made, derived from the RNA 3 component of the CMV-S strain carrying the coat protein (CP) gene and the RNA 1,2 component of the CMV-D strain. The CMV-S CP gene (AF063610) used in this study was obtained from full–length cDNA copies of CMV-S genomic RNA 3 (pCMV3S) of 2078 nt (the gift of Marie Tousignant, Agricultural Research Service, U.S.D.A., Beltsville, MD 20705, USA). The R9 mimotope nucleotide sequence was inserted in position 529 of the CP gene, as described by Natilla et al*.* [4], and the resultant plasmid served as template for in vitro generation of the corresponding CMV-S chimeric RNA 3.

One μg of DNA template was then used in a 20 μl volume reaction for synthesizing capped transcripts using the T7 mMessage mMachine^TM^ Kit (Ambion Europe LTD, Cambridgeshire, U.K.). Before transcription, the template was linearized with *Sma* I. The in vitro CMV-S RNA 3 transcript was then supplemented with the other two CMV genomic RNAs (RNA1,2/RNA3 1:2) deriving from CMV-D, to obtain the chimeric R9-CMV. A final RNA concentration of 0.5 μg/μl in 50 mM potassium phosphate, pH 7.0, was used to inoculate *Nicotiana tabacum* cv. Xanthi plants at the four-leaf stage. To verify the R9-CMV ability to spread systemically in the host, as well as to demonstrate that the R9 mimotope was exhibited in the planned exposed position, tissues were analyzed by RT-PCR, Western blot, ELISA and electron microscopy 10 days after inoculation, as previously described [4]. R9-CMV particles were purified and quantified by measuring the optical density of the virus suspension at 260 nm. Virus extraction yielded an average of 10 mg/100 g of fresh tissue.

CMV-D/S purified particles were used as controls in all the experiments.

### Patients

Forty untreated patients with chronic HCV infection (23 males and 17 females; mean age 51 years, range 27–65 years), admitted to the Department of Internal Medicine, Bari University Hospital, were enrolled in the study after giving informed consent. All patients were positive for HCV-RNA by polymerase chain reaction (PCR), had had abnormal alanine aminotransferase (ALT) serum levels for at least 6 months before inclusion in the study and had histopathological confirmation of chronic hepatitis. Exclusion criteria included alcoholism, use of hepatotoxic drugs, clinical and/or histological evidence of autoimmune hepatitis, inherited metabolic disorders and co-infection with other hepatotropic viruses (i.e., HBV and HDV). The HCV genotype was determined by Inno-Lipa HCV II (Innogenetics N.V., Ghent, Belgium), that allows genotyping of the 6 major HCV types and their most common subtypes. With this approach we found genotype 1b in 26 patients, 2a/2c in 11 patients and 3a in 3 patients. However, no differences in terms of apoptotic phenomena were observed among patients with regard to their histopathological pattern or viral genotype.

Twenty HCV negative healthy donors were included in the study as controls.

### Cell Cultures

PBMC were isolated from heparinized venous blood by Lympholyte (Cedarlane Laboratories, Hornby, Ontario, Canada) density gradient centrifugation. The cell suspensions recovered at the interface were washed and resuspended in RPMI 1640 (Sigma Chemical Co., Milan, Italy) supplemented with penicillin (200 IU/ml), streptomycin (100 μg/ml), L-glutamine (2 mM) and 10% heat inactivated fetal calf serum (FCS) (complete medium). The monocyte concentration in PBMC suspensions was approximately 15%, as evaluated by cytochemical (non-specific esterase) criteria.

PBMC (2 × 10^5^ cells/well; 1 × 10^6^ cells/mL) were incubated for 10 days at 37°C 5% CO_2_ in 96-well round-bottom microtiter plates (Corning Costar, Milan, Italy) in the presence of medium alone (Not Stimulated cells—NS), synthetic peptide R9 (10 μg/mL), R9-CMV (5 μg/mL), CMV-D/S (5 μg/mL) or, where indicated, Thapsigargin (TG, 2 μM).

PBMC were recovered daily in pilot experiments, and then after 48 h (specifically with reference to experiments in which TG was used), 3 days (T3) or 7 days (T7) of culture, to be analyzed for apoptotic events.

Where indicated, cell cultures were supplemented with purified caspase-8 (Z-IETD-FMK) or caspase-9 (Z-LEHD-FMK) inhibitors (5 μM/well) (Alexis Biochemicals; Vinci-Biochem, Florence, Italy), and cell apoptosis, as well as the activity of the inhibitor-free caspase, were evaluated after 3 days of incubation. For Western blot analysis, PBMC (5–8 × 10^6^ cells/flask, 1 × 10^6^ cells/mL) were cultured for 48 and 72 h at 37°C 5% CO_2_ in 25 cm^2^ flasks (Corning Costar) in the presence of R9-CMV (5 μg/ml), TG (2 μM) as stimulants, or medium alone.

All reagents were LPS-free, as assessed by the Limulus amebocyte lysate assay (PBI International, Milan, Italy).

### Cytofluorimetric Analysis of Cell Apoptosis

PBMC apoptosis was measured by flow cytometry using the Annexin V-FITC/7-AAD kit (provided by Beckman Coulter Inc., Miami, FL, USA), following the manufacturer’s instructions. Annexin V binds specifically to phosphatidylserine (PS), a phospholipid usually located in the inner leaflet of the plasma membrane that, in the early phase of apoptosis, becomes exposed at the cell surface and acts as a specific signal for recognition and removal of apoptotic cells by macrophages. Instead, 7-AAD is a DNA specific viability dye recognizing necrotic cells.

PBMC recovered from culture plates were washed with cold phosphate-buffered saline (PBS) and resuspended in 0.5 mL of binding buffer (Hepes 10 mM, NaCl 140 mM, CaCl_2_ 2.5 mM pH 7.4) in 2 mL conical bottom tubes. Cell suspensions were stained with Annexin V-FITC/7AAD at saturating concentrations, [Phycoerythrin (PE)-Texas Red] (ECD)-conjugated anti-CD3 and PE-labeled anti-CD19 monoclonal antibodies (mAbs) (all purchased from Beckman Coulter Inc.) on ice for 15 min in the dark. Then 400 μl of ice-cold Binding Buffer were added, cell preparations were analyzed within 30 min on a flow cytometer/cell sorter COULTER Epics Elite (Beckman Coulter Inc.) equipped with a 15 mm air-cooled, 488 nm argon-ion laser. Gating on physical parameters (forward- and side-scatter) was used to define lymphocytes and exclude cell debris and clumps. In each experiment, 15000 events were analyzed.

### Detection of Active Cellular Caspases by a Cytofluorimetric Technique Based on a FLuorochrome Inhibitor of Caspases (FLICA)

To study active cellular caspases, we used a method based on cell permeable, non-cytotoxic and fluorochrome-labeled inhibitors of caspases (FLuorochrome Inhibitors of CAspases—FLICA) that, once inside the cell, bind covalently to the respective active caspase, labeling it and inhibiting any further enzymatic activity. The complex caspase-inhibitor is retained within the cell, while any unbound reagent diffuses out of the cell and is washed away, so that the fluorescent signal is a direct measure of the number of active caspase enzymes present in the cell at the time the reagent is added. In this study, we used carboxyfluorescein (FAM)-labeled fluoromethyl ketone (FMK) peptide inhibitors of caspases emitting a green fluorescence that can be analyzed by flow cytometry.

Aliquots of PBMC under different experimental conditions (NS cells, cells treated with R9 peptide, R9-CMV or CMV-D/S) were collected after 3 and 7 days of culture, washed and incubated for 1 h in the dark at 37**°C** under 5% CO_2_ in the presence of the Poly-Caspases FLICA reagent (FAM-VAD-FMK), that irreversibly binds to many activated caspases (caspase-1, −3, −4, −5, −6, −7, −8 and −9), or with the specific inhibitors for caspase-8 (FAM-LETD-FMK) or caspase-9 (FAM-LEHD-FMK), following the manufacturer’s instructions (Immunochemistry Technologies, LLC, Bloomington, MN, USA). Cells were then washed twice with 1 mL of wash buffer (included in the kit) and resuspended in 600 μL of the same solution. Each sample was divided into two aliquots of 300 μL: one was directly analyzed by flow cytometry or, alternatively, fixed (by adding the fixative provided with the kit) and analyzed within 24 h; the other sample, prior to analysis, was supplemented with 1.5 μL of a ready-to-use solution of Propidium Iodide (PI) (250 μg/mL), which stained necrotic, dead and membrane-compromised cells, thus enabling live and dead cells to be distinguished within caspase-positive or -negative subsets. Samples labeled with FLICA and PI were directly read by flow cytometry, as the bicolor PI protocol did not allow cell fixing.

### Western Blot Analysis

Western blot was performed to evaluate PBMC levels of Bip/GRP78, GADD153, active caspase-12 and its precursor procaspase 12. PBMC, recovered after 48 and 72 h of culture as described above, were washed and resuspended in ice-cold lysis buffer containing 10 mM Hepes (pH 7.9), 10 mM KCl, 0.1 mM EDTA (pH 8.0), 0.2 mM EGTA (pH 6.0), 1 mM DTT, 1 mM phenylmethylsulfonyl fluoride, 10 μg/ml aprotinin, 10 μg/ml leupeptin, 1 mM sodium orthovanadate and 1 mM sodium fluoride for 15 min at 4**°C**. After addition of 10% Nonidet, samples were mixed and centrifuged, thereafter supernatants were mixed with sample buffer (2% sodium dodecyl sulphate [SDS], 7% glycerol, 0.72 M β-mercaptoethanol, 62.5 mM Tris–HCl, pH 6.8), heated at 100**°C** for 5 min and then frozen at −80**°C** until use. Samples were subjected to 10% polyacrylamide gel electrophoresis, after which proteins were electrophoretically transferred from the gel onto a nitrocellulose membrane in a buffer containing 25 mM Tris, 192 mM glycine and 20% methanol at 5.5 mA/cm^2^ for 30 min at room temperature. Residual binding sites on the membrane were blocked by incubating the membrane in blocking buffer (Tris-buffered saline pH 7.6 with 0.1% Tween 20 and 5% non-fat dry milk) for 1 h at room temperature under gentle agitation. After washing in Tris-buffered saline containing 0.1% Tween 20 (TBST), the membrane was incubated at 4**°C** overnight with rabbit polyclonal Abs anti-BiP/GRP78 (1 μg/ml), anti-caspase-12 (2 μg/ml), anti-procaspase-12 (1 μg/ml) or mouse monoclonal Abs to GADD153 (1 μg/ml) (all purchased from Abcam, Cambridge, UK). As secondary Abs, goat anti-rabbit IgG (Cell Signaling Technology, Beverly, MA, USA) or goat anti-mouse IgG (Abcam), both conjugated with horseradish peroxidase (1:10,000 in blocking buffer), were used and the membrane was kept at room temperature for 90 min under gentle agitation. The Ab complexes were visualized by the ECL Plus Western Blotting Detection Reagents (Amersham Biosciences Europe, Milan, Italy), following the manufacturer’s instructions, and subsequently densitometric analysis of each band was performed by means of the ImageMaster 1D Image Analysis Software (Amersham Biosciences Europe). Values were normalized to β-actin, assessed by stripping the membranes with the Restore^TM^ Stripping Buffer (Pierce, Rockford, IL, USA) and reprobing them with rabbit polyclonal anti-β-actin Abs (1:1000) (Santa Cruz Biotechnology, CA, USA).

To compare values from different gels, one lane of each gel was loaded with a control sample. After transfer and blocking, the membrane was cut and the control lane incubated with rabbit polyclonal Abs against β-actin (1:2000), chosen as house-keeping protein, before being processed together with the remaining part of the membrane as described above. Therefore, for each protein, values obtained from different experimental points were normalized to the β-actin of the control lane to offset any variations due to the different exposure of single membranes.

### Statistical Analysis

Statistical analysis was performed by Student *t* test. Statistical significance was set at a value of *P* < 0.05.

## Results

### Lymphocyte Spontaneous Apoptosis in Patients Chronically Infected with HCV

**Fig. 1:**
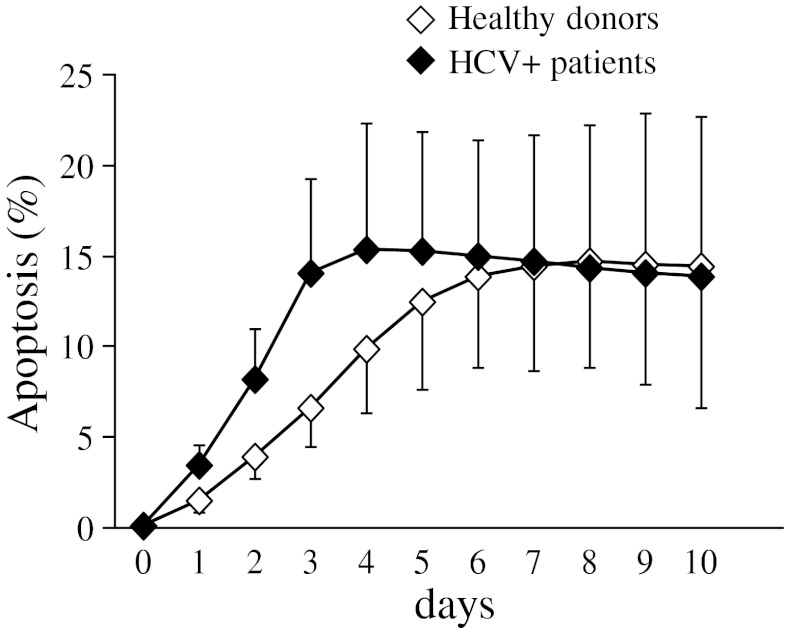
Time course evaluation of lymphocyte apoptosis occurring spontaneously in patients with chronic HCV infection and healthy donors (*n* = 4). Data are expressed as percentage of Annexin V^+^7-AAD^-^ lymphocytes (mean±SD)

The spontaneous apoptosis time course of peripheral blood lymphocytes was evaluated in all patients with chronic hepatitis C and compared with that exhibited by cells isolated from healthy donors. On the basis of pilot experiments, in which the proportion of apoptotic cells was measured daily for 10 days, data obtained at day 3 (T3) and day 7 (T7) of cell culture were considered the most representative (Figure 1). Simultaneous staining with the Annexin V-FITC/7-AAD kit, anti-CD19-PE and anti-CD3-ECD mAbs allowed differential analysis of B and T cell apoptosis.

**Fig. 2:**
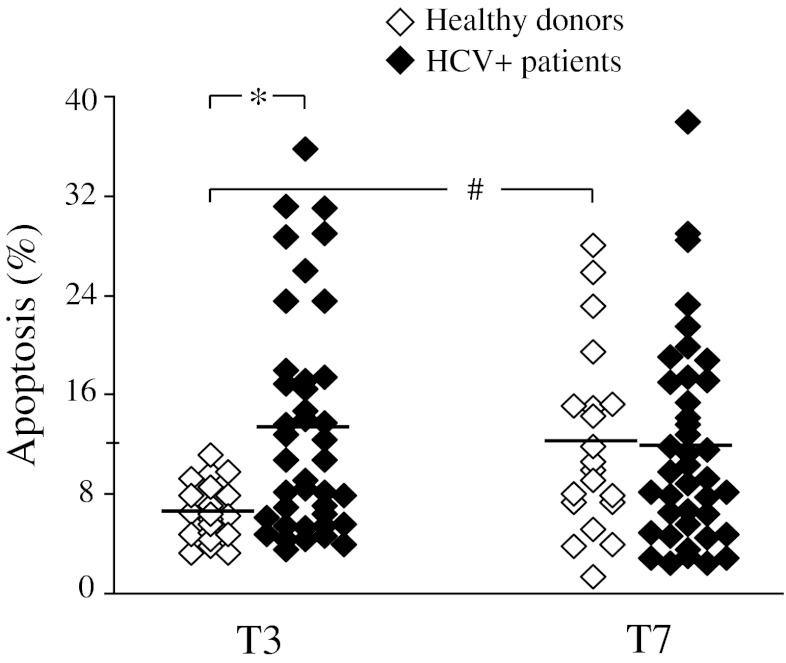
Lymphocyte spontaneous apoptosis in patients with chronic HCV infection (*n* = 40) and healthy donors (*n* = 20). Each dot represents individual donor’s data expressed as percentage of Annexin V^+^7-AAD^-^ lymphocytes at day 3 (T3) and day 7 (T7) of cell culture. Mean values are indicated by horizontal bars. Significance of HCV versus HD: *p < 0.01; significance of T7 versus homologous cells at T3: #*p* < 0.02

Lymphocytes from HCV+patients were found to undergo apoptosis quite rapidly, the percentage of apoptotic cells being significantly higher in the HCV+subset with respect to healthy controls already by 3 days of culture (mean±SD: 13.3 ± 8.9% and 6.6 ± 2.3% in patients and controls, respectively, *p* < 0.01) (Figure 2) . Then apoptotic events increased over time in the control subset, while they remained unchanged in the majority of HCV patients, so that the extent of the apoptotic process was comparable in patients and controls after seven days of cell culture (11.8 ± 8.2% versus 12.1 ± 7.4%, respectively, values being expressed as mean±SD) (see Figure 2).

**Fig. 3:**
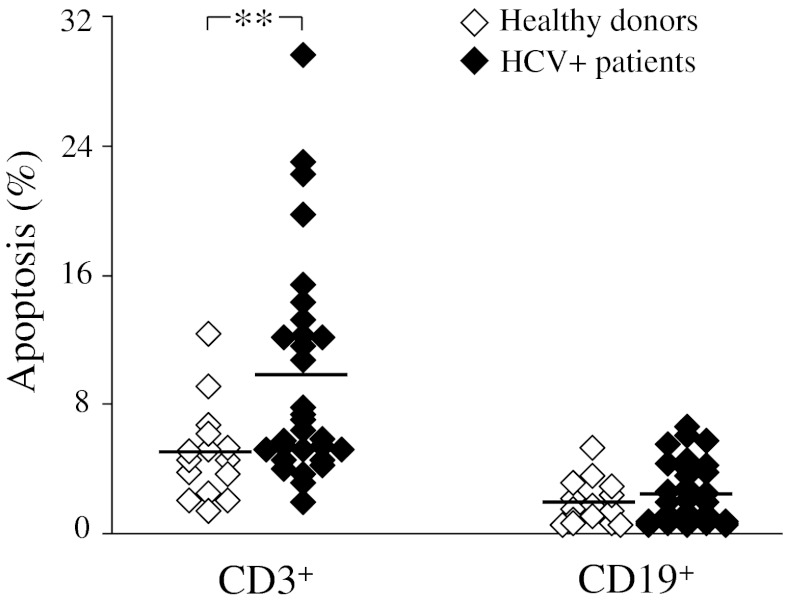
Spontaneous apoptosis in T and B lymphocytes from patients with chronic HCV infection (*n* = 30) and healthy donors (*n* = 15). Dots represent individual donor’s data expressed as percentage of Annexin V^+^7-AAD^-^ CD3^+^ or CD19^+^ lymphocytes after 3 days of cell culture. Horizontal bars indicate mean values. Significance of HCV versus HD: **p < 0.02

A detailed analysis within B and T cell populations highlighted that the above-described trend of early apoptotic phenomena seen in HCV patients was restricted to the CD3^+^ T cell subset (mean±SD of apoptotic events in patients and controls, respectively: 9.6 ± 6.8% and 5.0 ± 2.9% in CD3^+^ cells, *p* < 0.02; 2.4 ± 1.9% and 1.9 ± 1.4% in CD19^+^ cells) (Figure 3).

### Effects of the Chimeric Virus R9-CMV on Lymphocyte Apoptosis

Several reports have suggested that HCV may have significant effects on cell viability [8–13]. However, only few studies were focused on the effects of HCV molecules on the modulation of apoptotic events in the peripheral immune system. This prompted us to evaluate whether the HVR1-derived R9 mimotope might affect peripheral lymphocyte apoptosis. To this aim, a comparative analysis of spontaneous and R9-CMV-mediated apoptosis was carried out in PBMC from HCV patients and healthy donors. Cell apoptosis in the presence of the plant virus vector alone (CMV-D/S) or the synthetic peptide R9 was also assessed in parallel cultures.

**Fig. 4:**
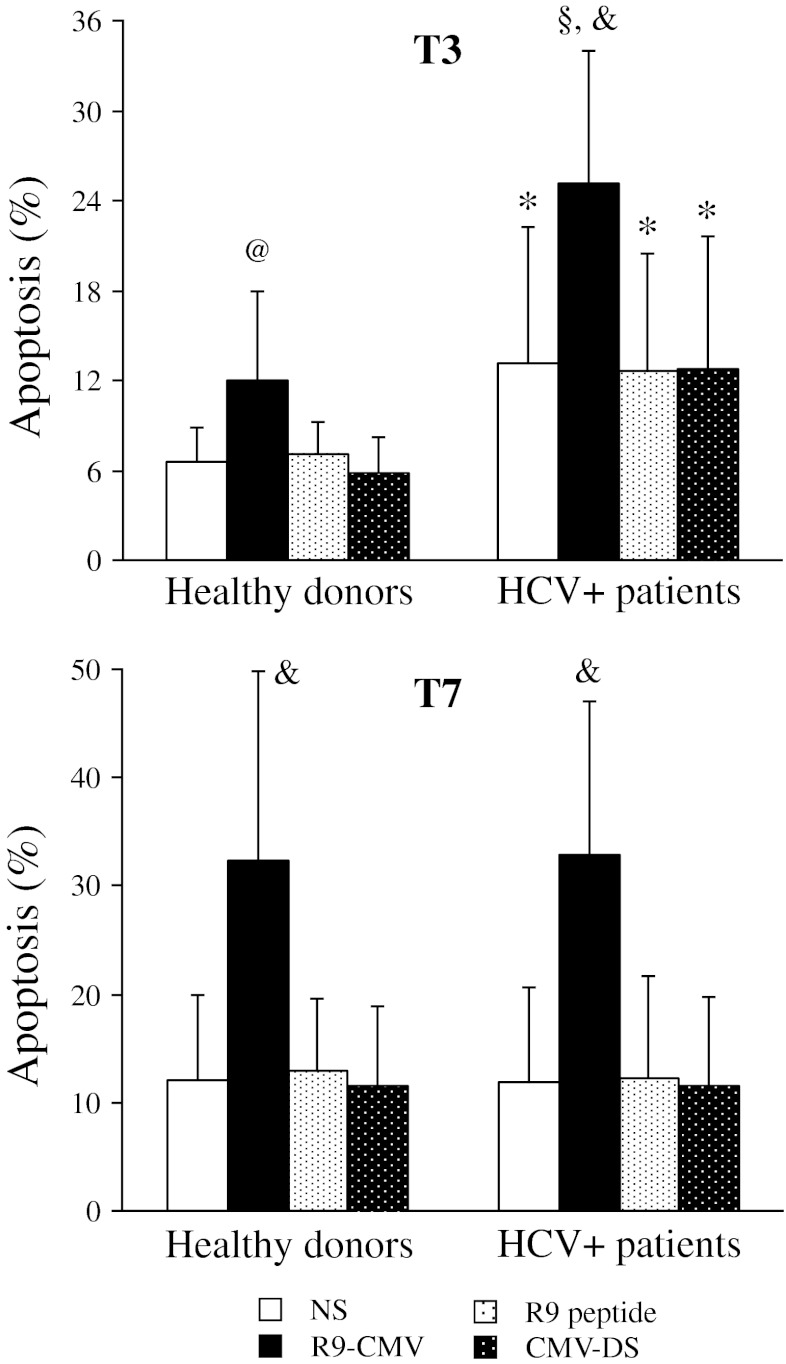
R9-CMV-induced apoptotic effects on lymphocytes from patients with chronic HCV infection (*n* = 40) and healthy donors (*n* = 20). Data are expressed as percentage of apoptotic lymphocytes (mean±SD) at day 3 (T3) and day 7 (T7) of cell culture in the absence of stimuli (NS) or after stimulation with R9-CMV, R9 peptide or CMV-D/S. Significance of HCV versus HD: *p < 0.01; ^§^ *p* < 0.001; significance of R9-CMV versus homologous NS cells: ^@^ *p* < 0.005; ^&^ *p* < 0.001

After 3 days, chimeric R9-CMV accelerated the apoptotic events occurring in untreated cells, with the lymphocytes from HCV patients being more susceptible to pro-apoptotic effects of the chimeric virus (see Figure 4). From then on, R9-CMV-triggered apoptotic phenomena increased in all subjects but, similarly to what was observed in unstimulated conditions, this effect was more pronounced in healthy controls. As a result, a difference in terms of apoptotic events between patients and controls was no longer observed after 7 days of R9-CMV-stimulation (Figure 4). Finally, as shown in Fig. 4, the synthetic peptide R9, as well as the vector alone (CMV-D/S), had negligible effects on spontaneous cell apoptosis in both patients and controls.

**Fig. 5:**
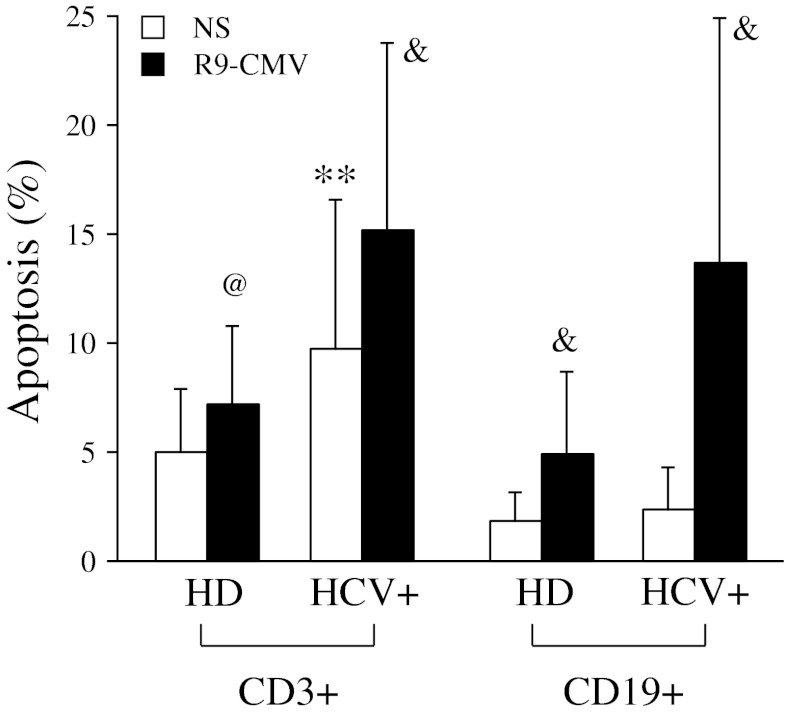
Effects of R9-CMV on T and B cell apoptosis in patients with chronic HCV infection (*n* = 30) and healthy donors (*n* = 15). Data are expressed as percentage of apoptotic CD3^+^ or CD19^+^ lymphocytes (mean±SD) after 3 days of cell culture in the absence or presence of R9-CMV as stimulant. Significance of HCV versus HD: **p < 0.02; significance of R9-CMV versus homologous NS cells: ^@^ *p* < 0.005; ^&^ *p* < 0.001

Analysis of lymphocyte subsets showed that although R9-CMV-mediated apoptosis occurred in both T and B cells, B lymphocytes, and especially those from HCV patients, were particularly sensitive to the pro-apoptotic effects of the chimeric virus (Figure 5).

### Effects of R9-CMV on Intracellular Caspase Activation in PBMC from HCV+Patients

The central component of the apoptotic process is a cascade of proteolytic enzymes known as caspases. To determine whether cell apoptosis induced by R9-CMV was associated with caspase activation, PBMC from HCV+patients were incubated with fluorochrome-labeled inhibitors of caspases (FLICA). By binding covalently to active caspases, FLICA label cells that have undergone apoptosis through caspase activation. Preliminary experiments showed that by using FAM-VAD-FMK, a generic probe for the detection of most caspases (caspase-1, −3, −4, −5, −6, −7, −8 and −9), a higher percentage of cells showing active caspases was found as a result of R9-CMV cell challenge as compared to unstimulated cells at both T3 and T7 (data not shown). By contrast, no significant differences in terms of FAM-VAD-FMK staining were detected between unstimulated cells and cells incubated with the synthetic R9-mimotope or the vector CMV-D/S throughout the incubation period (data not shown).

**Fig. 6:**
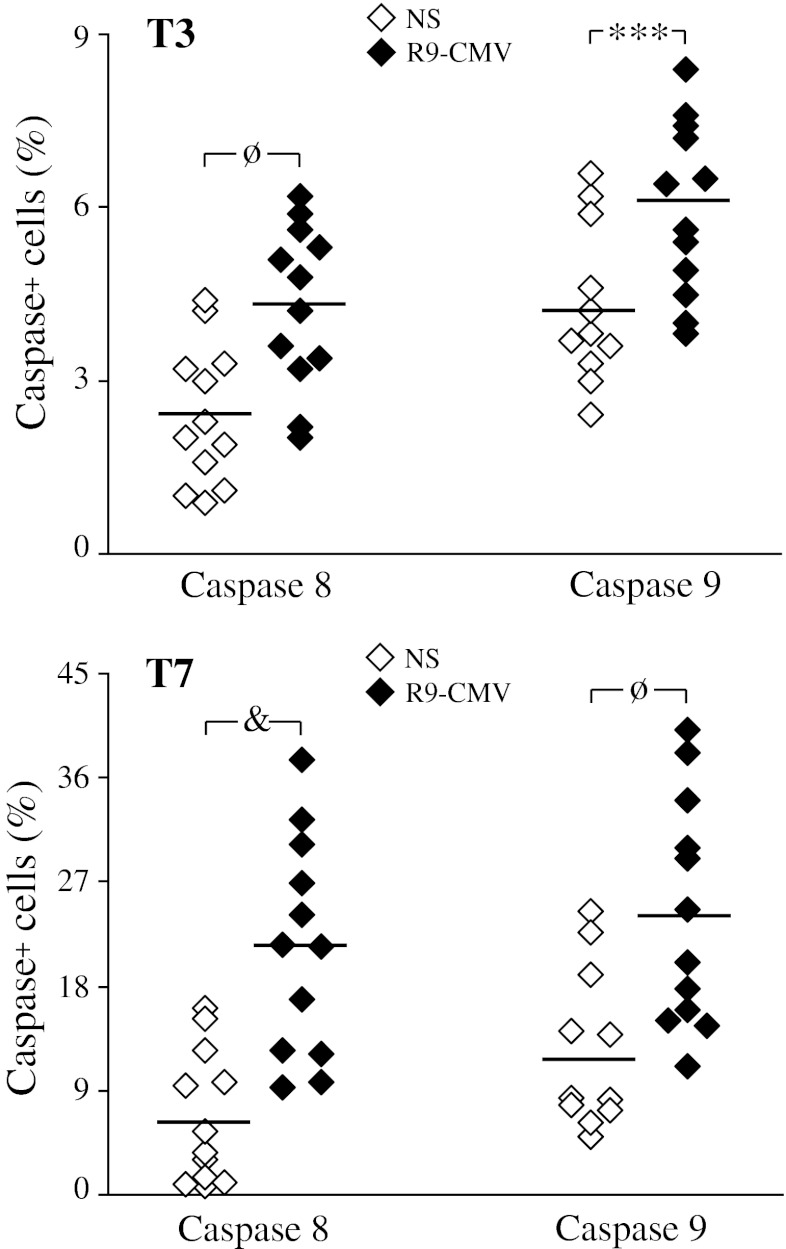
Effects of R9-CMV on caspase activation in lymphocytes from patients with chronic HCV infection (*n* = 12). Each dot represents individual donor’s data expressed as percentage of caspase-8 or −9 positive lymphocytes at day 3 (T3) and day 7 (T7) of cell culture in the absence of stimuli (NS) or after stimulation with R9-CMV. Mean values are indicated by horizontal bars. Significance of R9-CMV versus homologous NS cells: ***p < 0.01; ^ø^ *p* < 0.002; ^&^ *p* < 0.001

To assess whether R9-CMV-induced apoptosis occurred via a death receptor or a mitochondrial pathway, cells from HCV+patients were stained with FAM-LETD-FMK and FAM-LEHD-FMK, that primarily detect active caspases-8 and −9, respectively. We found that both caspases were significantly activated as a result of R9-CMV cell challenge, with caspase-8 activation being more impressive than caspase-9 activation at both T3 (caspase positive lymphocytes in unstimulated versus R9-CMV stimulated cells: 2.4 ± 1.2% and 4.3 ± 1.4% for caspase 8, *p* < 0.002; 4.3 ± 1.3 and 6.0 ± 1.5 for caspase 9, *p* < 0.01) and T7 culture time (unstimulated versus R9-CMV stimulated cells: 6.6 ± 5.7% and 21.2 ± 9.4% for caspase 8, *p* < 0.001; 11.8 ± 6.9 and 24.2 ± 9.9 for caspase 9, *p* < 0.002) (Figure 6).

**Fig. 7:**
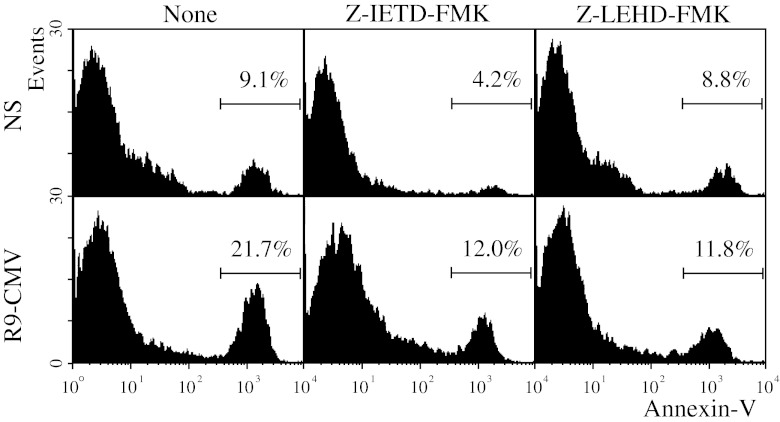
Effects of caspase inhibitors on spontaneous and R9-CMV-induced apoptosis in lymphocytes of patients with chronic HCV infection. PBMC from HCV+patients were incubated without (None) or with purified caspase-8 (Z-IETD-FMK) or caspase-9 (Z-LEHD-FMK) inhibitors. Data are expressed as percentage of Annexin V^+^7-AAD^-^ lymphocytes after 3 days of cell culture in the absence (NS) or presence of R9-CMV as stimulant, and are representative of 7 experiments with similar results

To further analyze the relationship between caspase activation and R9-CMV-triggered apoptosis, we tested the ability of specific purified inhibitors for caspases 8 and 9 to block both spontaneous and R9-CMV-induced apoptosis. As shown in Figure 6, spontaneous apoptosis was found to be significantly reduced only in the presence of the caspase-8 inhibitor Z-IETD-FMK, whereas R9-CMV-dependent apoptosis was inhibited in the presence of either the caspase-8- or the caspase-9 (Z-LEHD-FMK) specific inhibitor (Figure 7).

Finally, following R9-CMV stimulation, no significant modifications of active caspase-8 levels were detected as a result of selective caspase-9 inhibition, nor were differences in the activation of caspase-9 observed following selective inhibition of caspase-8 (data not shown).

### Effects of Chimeric R9-CMV on ER-stress and Caspase-12 Signaling in PBMC from HCV+Patients

There is increasing evidence that the endoplasmic reticulum might be implicated in the apoptotic execution. ER-associated pro-apoptotic molecules include caspase-12, Bap31 and GADD153 [25–28], whereas the chaperone protein BiP/GRP78 is an anti-apoptotic molecule that is strongly induced in response to ER-stress [26, 28].

**Fig. 8:**
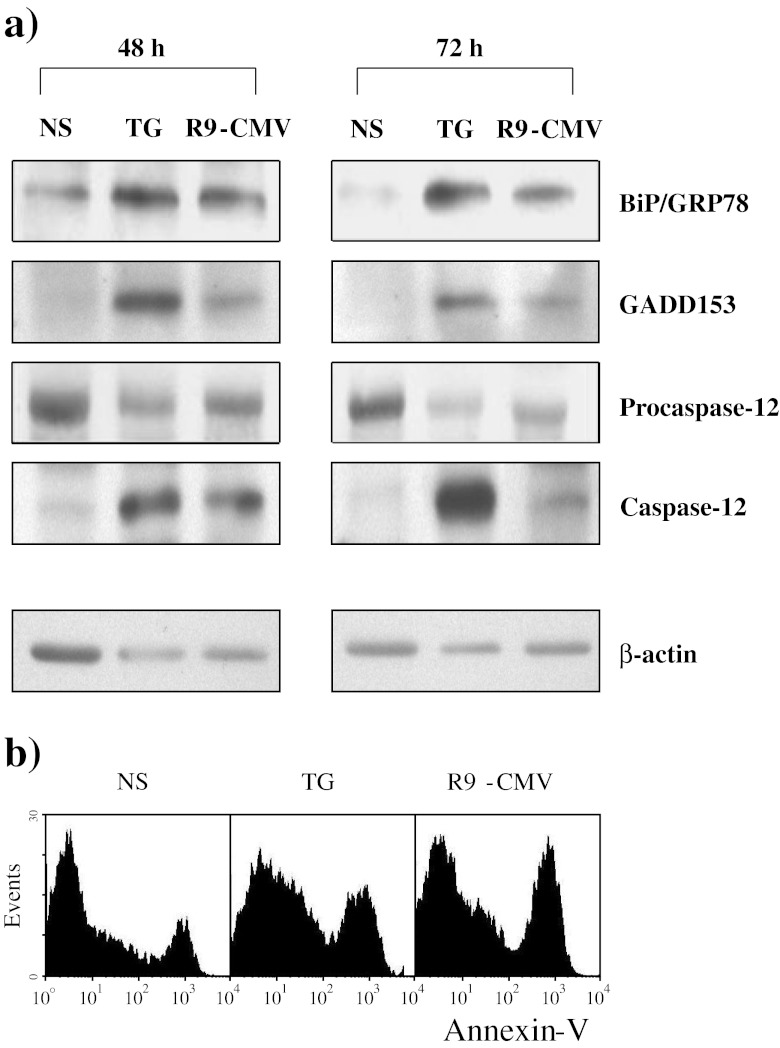
BiP/GRP78, GADD153, Procaspase-12 and active Caspase-12 expression in PBMC from patients with chronic HCV infection following Thapsigargin or R9-CMV cell stimulation. PBMC from HCV+patients were incubated with medium alone (NS), Thapsigargin (TG) or R9-CMV for 48 h or 72 h. Data are representative of 7 similar experiments. **a** Protein levels as determined by immunoblotting. An immunoblot performed by stripping the membrane and reprobing it with anti-β actin polyclonal Abs is also shown. **b** Annexin V^+^7-AAD^-^ lymphocytes after 72 h of cell culture

Considering that the ER is the main cellular compartment where the vital cycle of HCV takes place, we investigated its role in R9-CMV-induced apoptotic phenomena. To this aim, PBMC from HCV+patients were stimulated for 48 and 72 h with the chimeric virus and then the levels of BiP/GRP78 and GADD153 were assayed. Cells triggered with TG, which induces ER-stress through Ca^++^-adenosine triphosphatase inhibition [28], were considered as positive controls. Results demonstrated significant induction of both BiP/GRP78 and GADD153 as a result of R9-CMV cell stimulation (Figure 8). The effects of R9-CMV and TG on cellular levels of the enzyme inactive precursor procaspase-12, as well as of the active form of caspase-12, were also assessed. Interestingly, both stimulants gave rise to an increase in active caspase-12 that was paralleled by a progressive consumption of its precursor (Figure 8). All protein changes paralleled apoptotic events at 48 h (data not illustrated) and 72 h (Figure 8), as assessed by Annexin V staining of cells maintained under the same experimental conditions.

## Discussion

The role of apoptosis in HCV-related disease is still far from clear. Several aspects need to be better clarified, including the kinetics and extent of apoptotic processes, the expression of pro-apoptotic mediators during acute and chronic phases of infection, as well as their potential links to virus persistence.

In the current study, the early spontaneous apoptotic death exhibited in vitro by T cells from HCV+patients is likely a direct effect of the virus, exerted to escape immunological defenses or enhance its pathogenicity. An increased apoptotic T cell death has previously been described in chronic HCV-infected patients [29, 30], but only when in vitro “activated” peripheral T cells or T lymphocytes co-cultured with mitogen-activated monocytes were analyzed. The time course approach to the evaluation of T cell spontaneous apoptosis adopted in this study has disclosed this HCV pro-apoptotic effect also on resting T lymphocytes.

The chimeric plant virus R9-CMV has already been demonstrated to up-regulate HCV-specific immune responses [6, 7]. On these bases, it was of particular interest to use this chimeric system to study possible HCV HVR1 modulating effects on peripheral lymphocyte apoptosis. We found that, unlike the purified form of the R9 peptide, R9-CMV exhibited an early pro-apoptotic action in PBMC cultures from both patients and controls. Such an effect is clearly ascribable to the R9 residue expressed by the chimeric virus, as the pseudorecombinant CMV-D/S produced only negligible variations of lymphocyte apoptosis. The R9-CMV-mediated apoptotic effect observed in cells from healthy subjects is in line with evidence relating hepatocyte apoptosis with a cytopathic effect of HCV proteins [31, 32]. This further suggests that the glycoprotein E2, from which the synthetic R9 mimotope is derived, might be the mediator of the liver damage directly induced by the virus. On the other hand, the possibility that E2-induced lymphocyte apoptosis contributes to allow HCV to escape the host immune surveillance cannot be ruled out. In this regard, it should be stressed that R9-CMV-mediated effects, even if evident in both patients and controls, were more pronounced in HCV-infected subjects, thus indicating the ability of R9 mimotope, repeatedly expressed on this peculiar vector, to stimulate a memory immune response in the infected host.

In the last few years, it has been widely demonstrated that the apoptotic process involves a family of conserved intracellular proteases, called caspases, present in the cytosol of most cells as inactive proenzymes. During the execution phase, the concerted action of molecular and biochemical steps leads to the activation of common effectors or downstream caspases, which dismantle the cell by sequential cleavage of nuclear and cytoplasmic substrates [33, 34]. Two major pathways are known to activate these effector caspases: one involves death factors and death receptors (the “extrinsic” pathway), recruiting an intracellular death complex that activates a class of apical caspases, most notably caspase-8; the “intrinsic” pathway is related to mitochondrial dysfunction which, in turn, activates a different initiator caspase, caspase-9, also localized in the cytoplasm. Thereafter, the pathways converge in the activation of downstream caspases, such as caspase-3, −6 or −7 [33, 34].

By using a generic probe for the detection of most intracellular active caspases, we have shown that lymphocyte stimulation with the chimeric plant virus R9-CMV, but not with the synthetic R9 mimotope or with the empty vector CMV-D/S, was associated with caspase activation. Moreover, although both active caspase 8 and 9 were detected as a result of incubation with the chimeric virus, a predominant role of caspase-8 might be hypothesized, based on the evidence of a greater increase in the levels of active caspase-8 rather than caspase-9, in stimulated versus unstimulated cells. These results, together with data from our previous reports depicting the R9-CMV property to induce a TCR-mediated T cell activation and to stimulate a dominant Th-1-driven immune response [6, 7], led us to hypothesize a novel mechanism underlying the complex interaction between HCV and the infected host. Activation-induced cell death (AICD) is regarded as a major homeostatic mechanism in the immune system. This particular apoptosis pathway is thought to result from repeated T cell stimulation by persistent antigens, with the consequent engagement of death receptors and activation of caspase-8 [35, 36]. While interleukin-2 has been shown to sensitize T cells to AICD by enhancing Fas ligand expression, interferon-γ seems to act through the induction of caspase-8 expression downstream of the Fas death receptor [37]. These data are consistent with the evidence that R9-CMV-dependent apoptotic effects occur in the absence of a significant increase in cell surface Fas expression and that neutralizing anti-Fas ligand antibodies fail to rescue R9-CMV-stimulated lymphocytes from apoptosis (Piazzolla G, Tortorella C, unpublished observations). Furthermore, they are particularly intriguing in the light of previous reports showing that caspase-8 activity is required for the establishment of T cell memory during viral and protozoan infections [38, 39] and that an immunodeficiency syndrome with defective lymphocyte activation occurs in humans as a result of a caspase-8 mutation abrogating enzymatic activity [40]. It might be argued that the R9 mimotope has the intrinsic property of regulating both the elimination of repeatedly activated T cells and the generation of long-lived memory CD8^+^ T cells through caspase-8 activation. This latter feature, together with the capacity of R9-CMV to enhance Ag-specific CD8^+^ immune responses [6], opens up new prospects in the sense that they may offer both a novel vaccination strategy against HCV and a tool for boosting the effectiveness of such vaccines.

Caspase-9 activation, also triggered by R9-CMV, is another interesting aspect. By supplementing cell cultures with specific inhibitors for caspase-8 or caspase-9, we found that spontaneous apoptosis was inhibited only in the presence of the caspase-8 inhibitor Z-IETD-FMK. Conversely, both Z-IETD-FMK and Z-LEHD-FMK, the specific inhibitor of caspase-9, were found to be efficacious in decreasing R9-CMV-dependent apoptosis. In our experimental conditions, a crosstalk between the two main apoptotic pathways as a result of R9-CMV cell stimulation seems unlikely as we did not detect a significant reduction in caspase-9 activation following specific inhibition of caspase-8, nor a decrease in levels of active caspase-8 as a result of caspase-9 inhibition. The connection between R9-CMV and caspase-9 activation might rather be provided by a third subcellular compartment implicated in apoptotic execution, namely the endoplasmic reticulum. Alterations in calcium homeostasis are known to cause a calcium-dependent disruption of ER function, referred to as “ER-stress”, an event that may lead to apoptosis via caspase-9 and caspase-12 activation [41, 42]. In this regard, it has been reported that HCV E2-mediated cross-linking of CD81 lowers the TCR-mediated T cell activation threshold of naive and antigen-experienced T cell subsets, thereafter inducing cell calcium influx [43]. Likewise, it is plausible that the R9 mimotope expressed on the surface of the chimeric CMV in an ordered array of hundreds of molecules empowers the chimeric virus to cross-link lymphocyte CD81 and to induce cell calcium influx. The consequent elevation of intracellular calcium levels might thus trigger “ER-stress” ultimately leading to apoptosis. Our evidence that B lymphocytes, which are known to express high levels of CD81 on their surface, were particularly sensitive to R9-CMV-mediated apoptosis is consistent with this view. The role of ER-stress in our model was then confirmed by pilot experiments showing a significant induction of the ER-associated pro-apoptotic molecule GADD153, as well as of the ER-chaperon Bip/GRP78 protein, following R9-CMV lymphocyte treatment. In particular, Bip/GRP78 is a key mediator of the unfolded protein response (UPR), the adaptive program activated by cells to alleviate ER-stress, so the levels of this protein serve as an indicator of ER-stress [28, 44]. Interestingly, Bip/GRP78 levels measured in lymphocytes from HCV+patients following stimulation with the chimeric plant virus were similar to those assessed in cell cultures treated with TG, which is known to induce ER-stress through changes in intracellular calcium concentrations [28]. In keeping with these findings, a significant increase in active caspase-12 levels and a parallel consumption of its precursor procaspase-12 were detected both in R9-CMV- and TG-stimulated cell cultures under the same experimental conditions, this suggesting the possible role of caspase-12 as an effector caspase of the apoptotic pathway triggered by R9-CMV.

In conclusion, our results support the feasibility of producing a plant-derived Ag-presenting system to strengthen host defences against persistent pathogens. Particularly interesting and worthy of further investigation is the R9-CMV ability to stimulate a TCR-mediated T cell activation and, at the same time, to enhance a caspase-8-dependent immunological memory towards HCV. The role of R9-CMV in the modulation of ER-mediated cell apoptosis, whose importance in HCV infection is still largely unexplored, opens up new prospects for clarifying mechanisms involved in the pathogenesis of HCV-induced tissue damage and/or in the persistence of the virus.
